# Dynamic Contrast-Enhanced Imaging as a Prognostic Tool in Early Diagnosis of Prostate Cancer: Correlation with PSA and Clinical Stage

**DOI:** 10.1155/2018/3181258

**Published:** 2018-09-19

**Authors:** Xingchen Wu, Petri Reinikainen, Mika Kapanen, Tuula Vierikko, Pertti Ryymin, Pirkko-Liisa Kellokumpu-Lehtinen

**Affiliations:** ^1^Department of Oncology, Tampere University Hospital, Tampere, Finland; ^2^Faculty of Medicine and Life Sciences, University of Tampere, Tampere, Finland; ^3^Department of Radiology, Medical Imaging Centre, Tampere University Hospital, Tampere, Finland; ^4^Department of Medical Physics, Medical Imaging Centre, Tampere University Hospital, Tampere, Finland

## Abstract

**Background and Purpose:**

Although several methods have been developed to predict the outcome of patients with prostate cancer, early diagnosis of individual patient remains challenging. The aim of the present study was to correlate tumor perfusion parameters derived from dynamic contrast-enhanced magnetic resonance imaging (DCE-MRI) and clinical prognostic factors and further to explore the diagnostic value of DCE-MRI parameters in early stage prostate cancer.

**Patients and Methods:**

Sixty-two newly diagnosed patients with histologically proven prostate adenocarcinoma were enrolled in our prospective study. Transrectal ultrasound-guided biopsy (12 cores, 6 on each lobe) was performed in each patient. Pathology was reviewed and graded according to the Gleason system. DCE-MRI was performed and analyzed using a two-compartmental model; quantitative parameters including volume transfer constant (*K*^trans^), reflux constant (*K*_ep_), and initial area under curve (iAUC) were calculated from the tumors and correlated with prostate-specific antigen (PSA), Gleason score, and clinical stage.

**Results:**

*K* ^trans^ (0.11 ± 0.02 min^−1^ versus 0.16 ± 0.06 min^−1^; *p* < 0.05), *K*_ep_ (0.38 ± 0.08 min^−1^ versus 0.60 ± 0.23 min^−1^; *p* < 0.01), and iAUC (14.33 ± 2.66 mmoL/L/min versus 17.40 ± 5.97 mmoL/L/min; *p* < 0.05) were all lower in the clinical stage T1c tumors (tumor number, *n*=11) than that of tumors in clinical stage T2 (*n*=58). Serum PSA correlated with both tumor *K*^trans^ (*r*=0.304, *p* < 0.05) and iAUC (*r*=0.258, *p* < 0.05).

**Conclusions:**

Our study has confirmed that DCE-MRI is a promising biomarker that reflects the microcirculation of prostate cancer. DCE-MRI in combination with clinical prognostic factors may provide an effective new tool for the basis of early diagnosis and treatment decisions.

## 1. Introduction

Prostate cancer is the second leading cause of cancer-related death and the most frequently diagnosed male malignant disease in the Nordic countries [1]. Early detection of prostate cancer permits appropriate and timely management of the disease, and prognostic biomarkers can help clinicians to make a proper decision for treatment of individual patients and to avoid unnecessary treatments [2]. Although several methods have been developed to predict outcome of patients with prostate cancer, prognosis evaluation of individual patient remains challenging. Recent studies demonstrate that multiparametric magnetic resonance imaging (MP-MRI), consisting of T1-weighted, T2-weighted, diffusion-weighted imaging (DWI), and dynamic contrast-enhanced MRI (DCE-MRI), has emerged as a useful tool not only for localizing prostate cancer foci, but also for assessing tumor aggressiveness [3]. DWI allows to quantify the random motion of water molecules in tissue by means of apparent diffusion coefficient (ADC) measurements and provides information on tissue cellularity, tortuosity of extracellular space, and cell membrane integrity, thereby differentiating noncancerous and cancer lesions [4]. DCE-MRI is a relatively novel imaging modality that allows to measure properties of tissue microvasculature resulting from tumor angiogenesis and improving tumor detection and response assessment [5]. The most commonly used DCE-MRI parameter that reflects vascular permeability is the volume transfer constant (*K*^trans^) [6]. *K*^trans^ represents the rate at which the contrast agent transfers from the blood to the interstitial space, which indicates the tumor microcirculation and the surface infiltration area. In contrast, the reflux constant (*K*_ep_) reflects the rate at which the contrast agent transfers from the extravascular extracellular space back to the blood. The extravascular extracellular leakage volume fraction (Ve = *K*^trans^/*K*_ep_) predominantly reflects the percentage of contrast agent in the extravascular extracellular space [6]. In addition, the semiquantitative parameter initial area under curve (iAUC) is associated with tumor blood influx, perfusion, and interstitial space and represents the general tumor blood flow, overall perfusion, and tumor interstitial space index [6].

The aim of the present study was to correlate tumor perfusion parameters derived from DCE-MRI and clinical prognostic factors and further to explore if we can separate very early tumors from relatively advanced ones with DCE-MRI-derived parameters for decision making in early stage prostate cancer.

## 2. Materials and Methods

### 2.1. Patients

Seventy-one consecutive patients with histologically proven prostate adenocarcinoma were enrolled in our prospective clinical trials to develop hypofractionated image-guided and intensity-modulated radical radiotherapy. The study identifier at www.ClinicalTrials.gov is NCT02319239. The inclusion and exclusion criteria have been described in details in our previous publication [7]. Briefly, newly diagnosed adult patients with one or two of the intermediate-risk features (Gleason score 7, staging T2b-T2c, PSA 10–20 ng/mL) according to the National Comprehensive Cancer Network (NCCN) criteria [8], and patients were suitable for MRI examination. No patients received neoadjuvant or adjuvant hormonal treatment. The study was approved by the Ethics Committee of Tampere University Hospital (Nr. R14009), and all patients gave written informed consent prior to study entry. Patients underwent physical examination, digital rectal examination, and standard laboratory tests including serum prostate-specific antigen (PSA).

### 2.2. Histological Analysis

Transrectal ultrasound-guided biopsy (12 cores, 6 on each lobe) was performed in each patient. Six biopsy cores were embedded in one paraffin block. Pathology was reviewed and graded according to the Gleason system. Major criteria include an infiltrative glandular growth pattern and an absence of basal cells and nuclear atypia in the form of nucleomegaly and nucleolomegaly. The diagnosis was based on the microscopic appearance of slides stained using haematoxylin and eosin. In difficult cases, basal cell absence has been confirmed by immunohistochemical stains for basal cell markers.

### 2.3. Multiparametric MRI Acquisition

Multiparametric MR imaging was acquired using a 3 Tesla MR System (Siemens Trio-Tim, Erlangen, Germany) with a combination of 6-channel body matrix coil and 6 elements of 24-channel spine matrix coil positioned around the pelvis to cover the prostate. Tri-planar T2-weighted turbo spin echo images from below the prostatic apex to above the seminal vesicles were obtained. DWI was acquired with a single-shot echoplanar sequence on the axial plane using three *b* values (50, 400, and 800 s/mm^2^) and with the same orientation and location used to acquire axial T2-weighted images. DCE-MRI was performed with axial T1-weighted 3D volumetric interpolated breath-hold examination (VIBE) sequence that covers the entire prostate in consecutive sections. To determine the T1 relaxation time in the tissue before the arrival of contrast agent, the DCE-MRI included two precontrast 3D VIBE imaging sequences that had different flip angles (2° and 13°). These sequences were followed by a DCE series on the axial plane after gadolinium (Gd)-DOTA (0.2 ml/kg Dotarem®) injection, with a temporal resolution of 8 seconds and an acquisition time of 4 minutes 40 seconds. The contrast agent was administered using a power injector (Medrad Spectris Solaris EP, Bayer Medical Care Inc, PA, USA) followed by a 20 ml saline flush injection at a flow rate of 2.5 ml/s. To minimize postbiopsy artifact, MRI was performed 6–10 weeks after the prostate cancer confirmation by biopsy. For imaging parameters, see Table 1.

### 2.4. MR Image Analysis

All MR images were reviewed and analyzed on a syngo Multimodality Workplace (Siemens Healthcare). Voxelwise MRI signal enhancement time curves were fitted according to a pharmacokinetic model using Tissue 4D software (Siemens Healthcare). First, a motion correction has been performed, which registered all volumes of the time series to a user-selected reference volume to reduce the effect of patient and physiological motion during the DCE image acquisition. After the registration of the morphological image and the precontrast image, an oval-shaped or irregular-shaped region of interest (ROI) was drawn on the prostate cancer foci. ROIs were drawn in early enhancing region of DCE-MRI and with the DWI b800, ADC map, and T2-weighted image as references. T1 map calculation of precontrast was a prerequisite for pharmacokinetic modeling. T1 fitting was restricted to pixels with values above a noise level value (>20), and the respective values were automatically calculated by the system as a function of the entered contrast agents. For the Tofts modeling [6], Tissue 4D provides arterial input function (AIF) that are modeled using a biexponential function with three different modes (fast, intermediate, and slow). The AIF was chosen according to the fast sampling method to calculate kinetic parameters [9]. Parametric maps were calculated, and *K*^trans^, *K*_ep_, Ve, and iAUC of the selected ROI were automatically estimated by the software.

The ADC value of each identified tumor lesion was measured directly on the parametric ADC maps. The ADC map was reviewed simultaneously with the corresponding high *b* value DW images, T2-weighted images, and precontrast T1-weighted images. The slice of the ADC map containing the largest tumor extent was selected for analysis, and a ROI was drawn in the center of the tumor excluding the tumor edges. The mean ADC value and the size of the selected tumor area were generated at the workstation and recorded for analysis.

A prostate cancer was defined on each MRI as follows: a hypointense region relative to the adjacent parenchyma on T2-weighted image; a region with a low ADC value relative to the adjacent parenchyma on the ADC map; and a region with early wash-in and wash-out of contrast medium relative to the adjacent parenchyma on DCE-MRI. Precontrast T1-weighted images were used to identify postbiopsy hemorrhage (as an area with high signal intensity) to rule out false-positive findings.

### 2.5. Statistical Analysis

Statistical analysis was performed with SPSS (version 23.0, SPSS Inc., Chicago, Illinois, USA). A two-sided nonparametric Mann–Whitney U test was used to compare the patients age, PSA, tumor size, ADC, *K*^trans^, *K*_ep_, Ve, or iAUC between the peripheral and transitional zone tumor groups, between Gleason score 3 + 3 and 3 + 4 groups, and between different clinical stages. Spearman's correlation coefficient was used to evaluate the correlation between tumor size, ADC, *K*^trans^, *K*_ep_, Ve, iAUC, Gleason score, and serum PSA; *p* values less than 0.05 were considered significant.

## 3. Results

### 3.1. Patient Characteristics

No suspicious lesion was found on MRI in 7 out of the 71 patients with a biopsy proven prostate cancer; two patients had no DCE images due to allergy to the contrast agent. Sixty-nine lesions were detected in the prostate of the remaining 62 patients (age: mean ± SD: 70 ± 5 years, range from 60 to 79 years). Ten patients had clinical stage T1c and 52 had T2 (16 in T2a, 8 in T2b, and 28 in T2c) tumors according to TNM classification for prostate cancer. The serum PSA value (mean ± SD) was 9.5 ± 3.7 ng/mL, with the range from 3.4 to 19.1 ng/mL.

### 3.2. Pathological Results

There were 19 patients with a Gleason score 3 + 3, 41 with a Gleason score 3 + 4, and 2 with a Gleason score 4 + 3 tumor.

None of the measured parameters, including patients' age, serum PSA, and DWI- and DCE-MRI-derived parameters, were different between Gleason score 3 + 3 and 3 + 4 tumor groups.

### 3.3. Tumor Location

The majority of the tumors were in the peripheral zone (52, 75%), and the other 17 tumors were in the transitional zone.

There was no significant difference of the patients' age, serum PSA, tumor ADC, *K*^trans^, or iAUC between the peripheral and transitional zone tumor groups (Table 2).

- The size of peripheral zone tumors (lesion number, *n*=52) was smaller than that of the transitional zone tumors (*n*=17) (0.68 ± 0.41 cm^2^ versus 0.93 ± 0.59 cm^2^; *p* < 0.05).
- *K*_ep_ was higher in the peripheral zone tumors (lesion number, *n*=52) than that of the transitional zone tumors (*n*=17) (0.59 ± 0.21 min^−1^ versus 0.49 ± 0.24 min^−1^; *p* < 0.05).
- Ve was lower in the peripheral zone tumors (lesion number, *n*=52) than that of the transitional zone tumors (*n*=17) (0.27 ± 0.08 versus 0.32 ± 0.07; *p* < 0.05).

### 3.4. DCE-MRI-Derived Parameters

**Figure 1:**
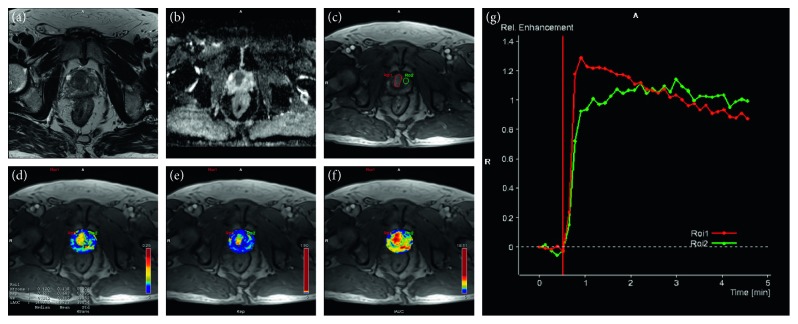
Transverse prostate MR images from a 69-year-old male patient with biopsy proven prostate cancer (Gleason score 3 + 4 and serum PSA 6.6 ng/mL): (a) T2-weighted image showing in the transitional zone a hypointense area without clear border; (b) ADC map: transitional zone hypointense region with a clear border, with ADC value of 0.75 × 10^−3^ mm^2^/s; (c) T1-weighted image early enhancement map: the enhanced region of interest 1 (ROI1, red line) corresponds to the tumor, and ROI 2 (green line) was selected from normal prostate tissue as healthy control; (d) *K*^trans^ map: ROI 1 *K*^trans^ 0.120 min^−1^ and ROI 2 *K*^trans^ 0.048 min^−1^; (e) *K*_ep_ map: ROI 1 *K*_ep_ 0.657 min^−1^ and ROI 2 *K*_ep_ 0.327 min^−1^; (f) iAUC map: ROI 1 iAUC 14.976 mmoL/L/min and ROI 2 iAUC 6.871 mmoL/L/min; (g) enhancement kinetics pattern from the two ROIs: the time-intensity curves were obtained from dynamic contrast-enhanced MRI. ROI1 showing a higher peak enhancement and an early wash-in and wash-out of contrast medium compared with ROI 2.

Prostate cancer showed earlier and more pronounced enhancement than surrounding normal prostate tissue (example Figure 1). Fifty-nine patients had perfusion MRI findings of at least one focal enhancing tumor in the prostate. In three patients, focal lesions were not obvious on the DCE images; all these 3 patients had clinical stage T2c tumors.

**Figure 2:**
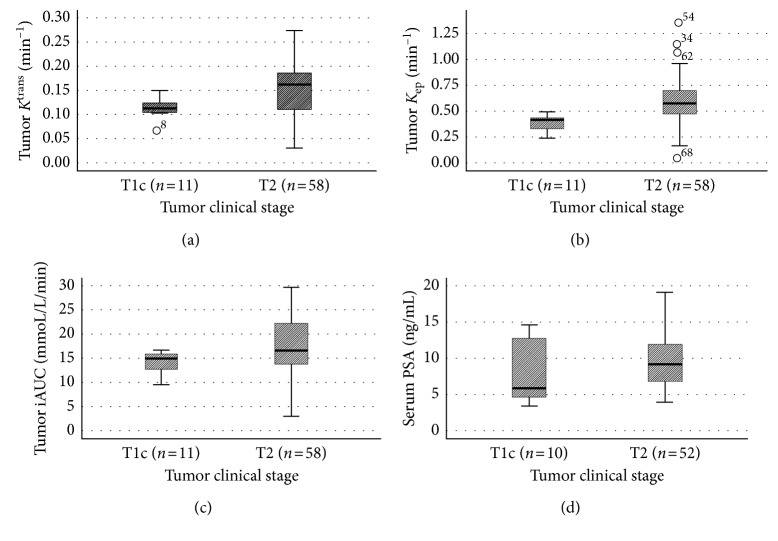
Comparison of tumor *K*^trans^, K_ep_, iAUC, and serum PSA level in patients with different clinical stages of prostate cancer. (a) *K*^trans^ (0.11 ± 0.02 min^−1^ versus 0.16 ± 0.06 min^−1^; *p* < 0.05), (b) *K*_ep_ (0.38 ± 0.08 min^−1^ versus 0.60 ± 0.23 min^−1^; *p* < 0.01), and (c) iAUC (14.33 ± 2.66 mmoL/L/min versus 17.40 ± 5.97 mmoL/L/min; *p* < 0.05) were all lower in clinical stage T1c tumors than that in clinical stage T2 tumors; (d) there was no significant difference of serum PSA between clinical stage T1c and T2 patients (8.2 ± 4.5 ng/mL versus 9.8 ± 3.6 ng/mL; *p*=0.151).

*K* ^trans^ (0.11 ± 0.02 min^−1^ versus 0.16 ± 0.06 min^−1^; *p* < 0.05), *K*_ep_ (0.38 ± 0.08 min^−1^ versus 0.60 ± 0.23 min^−1^; *p* < 0.01), and iAUC (14.33 ± 2.66 mmoL/L/min versus 17.40 ± 5.97 mmoL/L/min; *p* < 0.05) were all lower in the clinical stage T1c tumors (*n*=11) than that of the clinical stage T2 tumors (*n*=58) (Figures 2(a)–2(c)).

### 3.5. Serum PSA Value

There were no significant differences of the serum PSA levels between clinical stage T1c (*n*=10) and T2 patients (*n*=52) (8.2 ± 4.5 ng/mL versus 9.8 ± 3.6 ng/mL; *p*=0.151) (Figure 2(d)).

### 3.6. The Correlations between PSA and MRI Parameters

**Figure 3:**
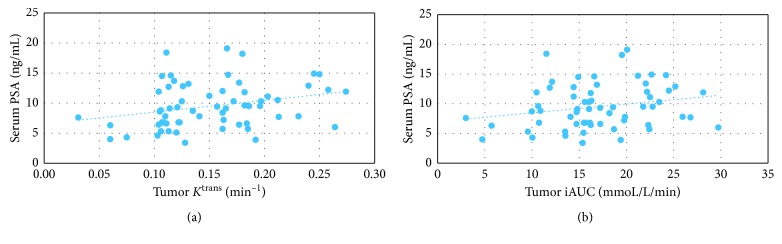
Correlations between serum PSA and DCE-MRI-derived tumor parameters in the 62 patients with prostate cancer. (a) Serum PSA correlated with tumor *K*^trans^ (*r*=0.317, *p* < 0.05); (b) Serum PSA correlated with tumor iAUC (*r*=0.258, *p* < 0.05).

Serum PSA correlated with both tumor *K*^trans^ (*r*=0.317, *p*=0.012) (Figure 3(a)) and tumor iAUC (*r*=0.258, *p*=0.043) (Figure 3(b)).

No correlation was found between serum PSA and tumor ADC value.

## 4. Discussion

A reliable diagnostic test should be able to provide an early prostate cancer diagnosis and minimize the amount of unnecessary biopsies or treatments. From this perspective, morphological MRI is a good candidate for prostate cancer investigation as it provides high-contrast and high-resolution images of the prostate. However, no single MRI sequence is sufficient to characterize prostate cancer. Each of the functional MR components has clinical advantages and limitations. Early promising data suggest that MP-MRI, which is performed concurrently with anatomical and functional techniques, is the most sensitive and specific imaging tool for lesion detection, characterization, and staging of prostate cancer [3]. Our study revealed a correlation between tumor *K*^trans^ and serum PSA in patients with early stage prostate cancer. This finding is consistent with previous publications [10, 11]. In addition, we detected a correlation between tumor iAUC and serum PSA. These may be explained by the altered vascular permeability of tumor microvessels and lymphatic system [12]. Neovascularity has been demonstrated to be a prerequisite for tumor growth and metastasis [13]. Abnormal angiogenesis in the tumor tissue lead to higher microvessel density, which is represented by leakage, twisted morphology, vascular wall expansion, and crosslinking [14]. Many scientists have suggested microvessel density as a prognostic and a predictive factor [15]. However, microvessel density measurement depends on the availability of postoperative tissue or biopsy materials, and it is a static assessment rather than information on vascular function. Therefore, there were controversial results between microvessel density and prostate cancer progression and grade [16]. In contrast, the distribution of Gd-DOTA in DCE-MRI is determined not only by microvessel density but also by vessel permeability and size of the extravascular extracellular space. DCE-MRI not only provides more details in tumor morphology but also allows to assess contrast agent kinetics and thus allows to improve detection and grading of prostate cancer.

DCE-MRI can be used to assess noninvasively the functional aspects of microcirculation of tissues. DCE-MRI relies on the fact that a bolus of contrast agent passing through the capillary bed is transiently confined within the vascular space before passing rapidly into the extravascular extracellular space at a rate determined by the permeability of the microvessels, their surface area, and blood flow [17, 18]. In DCE-MRI, the distribution of the contrast agent is repeatedly measured, allowing the evaluation of the tumor microcirculation in vivo and enabling the malignancy or benignancy of the tumor to be quantitatively distinguished [19]. Neoangiogenesis plays a vital role in the growth, progression, and metastasis process of prostate cancer [20, 21]. Microvessel density in prostatic carcinoma has also been shown to be an independent predictor of the pathological stage [13]. In consistent, we found that the tumor *K*^trans^, *K*_ep_, and iAUC were all lower in smaller tumors (T1c) than in larger local tumors (T2) in biopsy proven prostate cancer. To our knowledge, this is the first report that revealed DCE-MRI-derived parameters can separate very early stage tumors and relatively advanced tumors in clinically localized prostate cancer. Quantification of tumor angiogenesis by DCE-MRI may allow stratification of patients to type of treatment.

Serum PSA is elevated as a result of disruption of the prostatic architecture in the presence of prostate disease and injury, and PSA screening helps to diagnose prostate cancer earlier, at lower clinical stages and with lower Gleason score [22]. However, we did not find significant difference of the serum PSA levels between the tumors in clinical stage T1c and those with relatively extent diseases, for example, clinical stage T2. Serum PSA is not a specific marker for prostate cancer because of variable contribution to PSA from benign tissue and the nonlinear relationship between grade and PSA, which lead to overlap in PSA levels between different clinical stages as shown also in previous studies [23]. As a result, serum PSA level cannot be used alone to accurately predict disease extent for any individual patient. DCE-MRI play a role in conjunction with PSA for localizing suspicious lesions for biopsy, improving specificity, and identifying those tumors that are more likely to cause death if they are left untreated.

The Gleason score reflects the tumor aggressiveness and is an important predictor of outcome in patients with prostate cancer [2]. Correlation between the Gleason score and DCE-MRI-derived parameters may have been expected, because the Gleason scores have been shown to correlate with microvessel density measurements [13]. However, we did not detect any significant difference of the DCE-derived parameters between patients with Gleason score 3 + 3 and 3 + 4. The lack of differences may be explained by the heterogeneity of tumor tissues [24] and the histological sampling errors inherent in needle biopsy. Secondly, our patients were selected with one or two of the intermediate-risk features. Therefore, the differences of their disease extent/magnitude are relatively small compared with previous publications [10, 11].

Our study has a few limitations: firstly, the MRI was performed after biopsy. We were not sure, if the tumor ADC value and DCE parameters had been measured at the biopsy sites. Secondly, we were unable to evaluate the correlation between MRIs and histopathological features accurately because we did not obtain surgical specimens. There have been concerns about the probability of undergrading prostate cancer by biopsy due to tumor heterogeneity. Thirdly, all patients underwent needle biopsies before MRI examinations, implying that hemorrhagic or inflammatory changes caused by this procedure might have affected the MRIs. However, we excluded visible bleeding with the help of precontrast T1-weighted images, and the time interval between biopsy and MRI was long (6–10 weeks) enough for biopsy wound healing.

## 5. Conclusions

In conclusion, the present study has confirmed that DCE-MRI is a promising biomarker that reflects the microcirculation of prostate cancer. DCE-MRI-derived quantitative parameters in combination with clinical prognostic factors may provide an effective pretreatment diagnosis modality for early prostate cancer, especially for those with negative biopsy.

## Figures and Tables

**Table 1:** Sequence parameters for 3T multiparametric MRI with the body and spine matrix combination coil system.

Sequence	Pulse sequence	TR (msec)	TE (msec)	FA (°)	FOV (mm)	ACQmatrix	Slice/gap (mm)
Axial DWI, *b* = 50, 400, and 800 s/mm^2^	SE-EPI	3800	77	90	221 × 260	102 × 160	3.6/0
Axial T2W	TSE	4000	100	90	200 × 200	288 × 320	3/0.6
Sagittal T2W	TSE	5000	100	90	200 × 200	288 × 320	3/0.6
Coronal T2W	TSE	5000	100	90	200 × 200	288 × 320	3/0.6
Axial 3D^*∗*^	FLASH GRE	4.9	1.7	2 and 13	260 × 260	138 × 192	3/0
Axial 3D DCE	FLASH GRE	4.9	1.7	12	260 × 260	138 × 192	3.6/0

SE, spin echo; EPI, echo planar imaging; TSE, turbo spin echo; FLASH, fast low angle shot; GRE, gradient recalled echo; TR, repetition time; TE, echo time; FA, flip angle; ACQ matrix, acquisition matrix. ^*∗*^Sequence for the measurement of T1 relaxation time.

**Table 2:** Comparison of the 62 patients with peripheral and transitional zone prostate cancer (46 versus 16): age, tumor size, and DWI- and DCE-derived tumor parameters.

	Total *n*=69 mean ± SD	Peripheral *n*=52 mean ± SD	Transitional *n*=17 mean ± SD	*p* value
Age (years)	70 ± 5	70 ± 5	70 ± 4	0.974
PSA (ng/mL)	9.5 ± 3.7	9.7 ± 3.9	9.1 ± 3.3	0.552
Area of tumor (cm^2^)	0.74 ± 0.47	0.68 ± 0.41	0.93 ± 0.59	*0.037*
ADC (×10^−3^ mm^2^/s)	0.87 ± 0.16	0.89 ± 0.17	0.82 ± 0.13	0.259
*K* ^trans^ (min^−1^)	0.15 ± 0.05	0.15 ± 0.05	0.14 ± 0.06	0.743
*K*_ep_ (min^−1^)	0.57 ± 0.22	0.59 ± 0.21	0.49 ± 0.24	*0.048*
Ve	0.28 ± 0.08	0.27 ± 0.08	0.32 ± 0.07	*0.026*
iAUC (mmoL/L/min)	16.70 ± 5.69	17.26 ± 5.51	15.86 ± 6.21	0.626

PSA, prostate-specific antigen; ADC, apparent diffusion coefficient; *K*^trans^, volume transfer constant; *K*_ep_, reflux constant; Ve, extravascular extracellular leakage volume fraction; iAUC, initial area under curve; *n*, number of tumors.

## Data Availability

The data are available from the Medical Imaging Center of Tampere University Hospital.
